# Tension in Cancer

**DOI:** 10.3390/ijms17111910

**Published:** 2016-11-16

**Authors:** Stefanie Löffek, Claus-Werner Franzke, Iris Helfrich

**Affiliations:** 1 Skin Cancer Unit of the Dermatology Department, Medical Faculty, West German Cancer Center, University Duisburg-Essen, 45147 Essen, Germany; iris.helfrich@uk-essen.de; 2 German Cancer Consortium (DKTK), University Duisburg-Essen, 45147 Essen, Germany; 3 Department of Dermatology and Venerology, Medical Center, University of Freiburg, Hauptstraße 7, 79104 Freiburg, Germany; claus-werner.franzke@uniklinik-freiburg.de

**Keywords:** integrins, matrix stiffening, tumor progression, TGF-β, drug-resistance

## Abstract

Integrins represent a large family of cell receptors that mediate adhesion to the extracellular matrix (ECM), thereby modulating a variety of cellular functions that are required for proliferation, migration, malignant conversion and invasiveness. During tumorigenesis the conversion of a tumor cell from sessile, stationary phenotype to an invasive phenotype requires the ability of tumor cells to interact with their environment in order to transduce signals from the ECM into the cells. Hence, there is increasing evidence that changes in the composition, topography and tension of tumor matrix can be sensed by integrin receptors, leading to the regulation of intracellular signalling events which subsequently help to fuel cancer progression. The fact that intracellular signals perceived from integrin ligand binding impact on almost all steps of tumor progression, including tumor cell proliferation, survival, metastatic dissemination and colonization of a metastatic niche, renders integrins as ideal candidates for the development of therapeutic agents. In this review we summarize the role of integrins in cancer with the special focus on cancer therapies and the recent progress that has been made in the understanding of “integrin-induced tension in cancer”. Finally, we conclude with clinical evidence for the role of integrin-mediated mechanotransduction in the development of therapy-resistant tumors.

## 1. Introduction

The first integral membrane protein complex was discovered in 1986 by Tamkum and co-workers and was named “integrin” because this protein linked the extracellular matrix to the actin-cytoskeleton [1]. Since then, a tremendous amount of research has identified diverse and essential roles of integrins not only in embryonic development, tissue homeostasis, tissue repair and host defence but also in carcinogenesis. The participation of several members of the integrin family in cancer progression and invasion has made them an appealing target for the development of therapeutic agents, such as integrin antagonists and blocking antibodies that have been shown to disturb tumor cell invasion and cancer-induced angiogenesis [2,3,4,5]. However, in recent years, increasing evidence has revealed that physical features of the tumor environment, particularly matrix topology and stiffness, can alter the integrin-mediated intracellular signalling of tumor cells which may alter tumor cell behavior. Thus, increased stiffness of the adjacent tumor environment can regulate tumor malignancy as well as the drug-sensitivity of tumor cells by enhancing integrin-mediated mechanotransduction.

## 2. Integrins

### 2.1. Structure and Ligand Affinity of Integrins

Integrins are large, heterodimeric transmembrane proteins consisting of noncovalently associated α- and β-subunits [6,7]. So far, 18 α- and 8 β-subunits have been identified in humans, which generate 24 heterodimers with distinct ligand (Table 1) and signalling specificity. Generally, both α- and β-subunits consist of an approximately 700 amino acids (aa) long extracellular part which mediates binding to diverse extracellular matrix (ECM) proteins. The single-pass transmembrane helix of both type I oriented subunits is about 20 aa in length and the unstructured cytoplasmic tail is less than 75 aa (with the exception of the β4-subunit with a cytodomain of 1000 aa). Whereas the ligand specificity is mainly determined by the α-subunit (Table 1) the β-subunits bind to intracellular ligands that connect the integrin to signalling pathways and cytoskeletal networks [8,9]. Hence, the binding of extracellular and intracellular ligands by integrins permits a bi-directional transmission of mechanical force and biochemical signals across the plasma membrane [7].

As the tightly regulated integrin-ECM ligation is an important mechanism by which cells modulate integrin signalling, intensive research has been performed to elucidate the structural changes that determine the transition to different functional states. On the basis of structural and functional studies several reports suggest that integrin heterodimers can exist in different conformations that vary in their affinity for ligands. While the bent conformation is associated with low substrate affinity, extended conformation has been linked to high substrate affinity [10,11,12,13,14,15]. However, the exact dynamic equilibrium among different conformations remains controversial, as discussed in [11,14,16,17].

### 2.2. Integrin-Mediated Adhesion and Physical Forces

Cell adhesion mediated by integrins is a balanced combination of cell-matrix attachment and detachment and is crucial for the proper embedding and anchorage of cells within three-dimensional tissues. The major cell-matrix junctions in mammals are hemidesmosomes (HDs) and focal adhesions (FAs), both of which consist of transmembrane and intracellular protein components including integrin receptors that transmit signals in both directions across the membrane, outside-in and inside-out [7].

HDs are exclusively found on the basal membrane of epithelial cells where they provide cell-anchorage to the underlying basement membrane (BM). The intracellular side of HDs consists of a plaque of intracellular adapter proteins (bullous pemphigoid antigen 230, BP230 and plectin), which provides the link to the intracellular intermediate filaments [18]. For example, in skin keratinocytes, α6β4 integrin, in conjunction with transmembrane collagen XVII (also known as bullous pemphigoid antigen 180; BP180), mediates a strong binding to laminin-332, the major BM component of the skin. Consequentially, genetic ablation of either of these transmembrane proteins causes decreased dermal-epidermal adhesion leading to junctional epidermolysis bullosa (JEB) in humans and mice [19,20,21,22]. Although HDs are traditionally seen to be not involved in cell motility during wound healing or carcinoma invasion, almost all hemidesmosomal components, including α6β4 integrin and collagen XVII, were highly expressed at the leading edge of cutaneous wounds or the invasive front of squamous cell carcinoma [23,24,25]. Indeed there are several studies that demonstrate important functions of both molecules in the regulation of cell motility [23,26,27,28].

**Figure 1:**
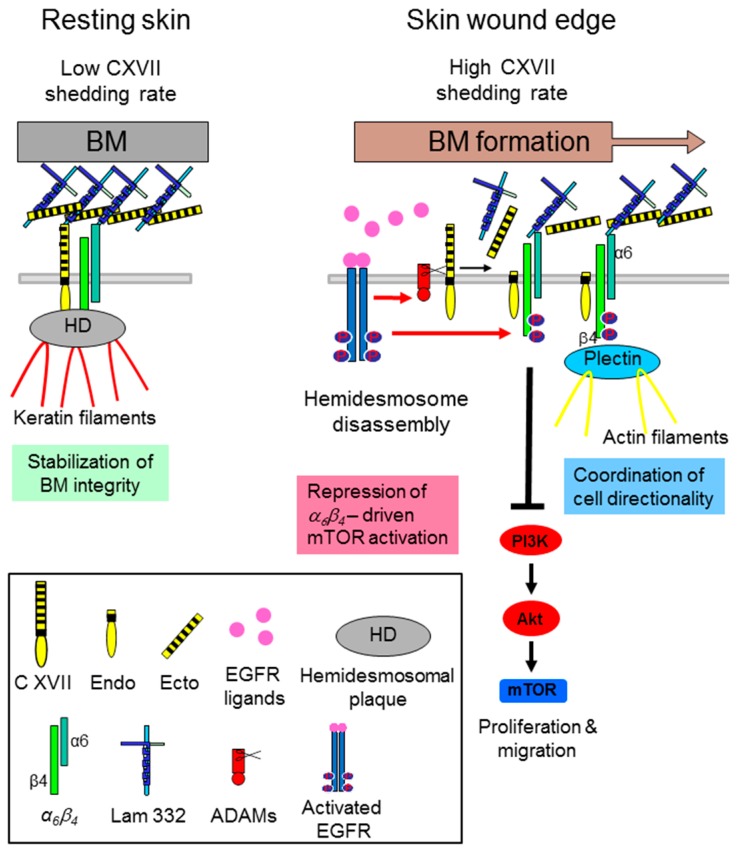
Collagen XVII (C XVII) ectodomain (Ecto) shedding modulates α6β4 integrin-driven motility and proliferation of wound keratinocytes. In resting skin collagen XVII and α6β4 integrin are part of the hemidesmosomal adhesion complexes and the continuously shed collagen XVII ectodomain (low shedding rate) is integrated into the basement membrane (BM). During cutaneous wound healing, when the hemidesmosomes (HDs) at the wound edges are disrupted via epidermal growth factor receptor (EGFR)-mediated phosphorylation of the β4-subunit, collagen XVII shedding is strongly induced. The released ecto- and endodomains have a repressive function on keratinocyte velocity and proliferation through dampening of α6β4 integrin-driven Akt/ mechanistic Target of Rapamycin (mTOR) pathway activation. While the released ectodomain is part of the newly formed BM surface topography, the membrane tethered endodomain stump seems to be involved in cell-intrinsic motility via formation of the front-to-rear polarity (Figure adapted from [23]).

Changes in laminin-332 deposition and processing are considered to promote invasion of skin cancer cells [29]. It has been shown that the deposition of laminin-332 directs integrin contacts to orchestrate polarization and linear migration of leading cells [30]. The laminin-332 receptors α6β4 integrin and collagen XVII seem to be intensively involved, because knockout of either one resulted in scattered laminin-332 deposition and disturbed directed migration in keratinocytes in vitro [26,31,32,33]. Our recent investigations revealed that, in particular, the ectodomain shedding of collagen XVII seemed to act as a dynamic modulator of directed keratinocyte motility by coordination of α6β4 integrin-laminin 332 interactions (Figure 1). Subsequently, shedding of collagen XVII appeared to have a repressive effect on keratinocyte hypermotility and proliferation through coordination of laminin-332 deposition and dampening of α6β4 integrin-driven phosphatidylinositol-4,5-bisphosphate 3-kinase (PI3K)/Akt/mTOR pathway activation [23].

Although FAs share the molecular organization of HDs, they differ in some aspects: (i) they are mainly found in highly motile cells; (ii) their turnover is highly dynamic and (iii) their intracellular linker proteins provide attachment sites for the actin-cytoskeleton. In general it is believed that the assembly of FAs is initiated by the binding of individual integrins to their ligands (Table 1), which results in integrin clustering and formation of nascent cell-ECM adhesion structures, also referred to as adhesome. The activation (high-affinity state) and clustering of integrin receptors is driven by the binding of activator proteins talin or kindlin to a conserved NPXY motif at the intracellular tail of the β-subunit of integrin receptors and this process is therefore called inside-out signalling [34]. The adhesome is composed of small GTPases and their modulators, scaffolding adapters (paxilin, kindlin) that form bridges between focal adhesion proteins as well as cytoskeletal-binding proteins (vinculin, α-actinin, talin) and catalytic adaptors (e.g., focal adhesion kinase (FAK), and integrin-linked kinase (Src)) that link the ECM adhesion site to the actin cytoskeleton and propagate signal transduction from adhesion sites, respectively [35].

The mechanical-force-dependent facilitation of actin polymerization (actin bundles and stress fibers) is a prerequisite for the maturation of FA and controlled by the Rho/Rho-kinase (ROCK) signalling pathway and requires the action of the actin regulatory proteins Arp2/3 complex, mammalian Diaphanous (mDia)-related formins and Ena/VASP [36]. Rho activated ROCK inhibits actin filament depolymerization and mediates myosin light chain (MLC) phosphorylation, hence conferring contractility on a cell [36]. Recently, the transcription factors YAP (Yes-associated protein) and TAZ (transcriptional coactivator with PDZ-binding motif, also known as WWTR1) have been suggested as key mediators in integrin-mediated mechano-transduction [37]. The nuclear localization of both factors seems to be regulated by contractile F-actin structures and maybe, by so far unidentified molecular effectors, depending on the cell type [37,38]. However, the detailed mechanisms and signalling hierarchy by which cytoskeletal tension regulates the translocation of YAP/TAZ await further evaluation.

The binding of the integrin receptor to ligands also activates several different pathways including for example FAK/Akt, integrin-linked kinase (ILK), Ras/MAPK/ERK kinase (MEK1/2)/signal–regulated kinases (ERK) 1/2 and c-Jun NH2-terminal kinase (JNK) [9]. This so-called outside-in signalling is very complex, cell type specific and highly dependent on the type of the integrin heterodimer. Interestingly, further signalling events within a cell have been discussed as a driver for this process. For example the β4-subunit can be phosphorylated at three serine residues (Ser-1356, Ser-1360, and Ser-1364) by pathways downstream of the epidermal growth factor EGF receptor (EGFR), e.g., ERK1/2 or by protein kinase C (PKC) [39,40,41]. Therefore, excessive EGFR signalling in tumor cells, seen in a wide variety of types of solid tumors, might have different impact on β4-subunit phosphorylation and subsequently also on integrin-mediated signalling. In striking contrast to the function of α6β4 integrin in basal keratinocytes (where the integrin is attached to the intermediate filaments) this receptor can instead be attached to the actin cytoskeleton in carcinomas were it stimulates tumor cell migration and invasion [42]. At this point we would like to emphasise that there are studies that have shown ligand-independent (anchorage-independent) integrin-function/signalling that might be relevant during metastatic dissemination [43].

## 3. Interfering Integrin-ECM Interactions as a Potential Therapeutic Strategy

Consistent with their essential role in cell adhesion to the extracellular matrix, dysregulation of integrin expression and their associated signalling pathways have been associated with pathological states such as carcinogenesis and the metastatic spread of tumor cells [44,45,46,47,48]. Although the above mentioned examples portray the complexity and dynamics of integrin-signalling, particularly in the context of carcinogenesis and tumor cell progression, interfering with integrin-ECM interactions appears to be an auspicious strategy for the development of therapeutic agents (Table 2). Nevertheless, the ability of some subunits—in particular β1 and αv—to form multiple heterodimers made this approach unexpectedly challenging.

Accumulating evidence suggests that tumor progression is initiated by so called “tumor-initiating cells” (TICs) and that the expression of distinct integrin subunits in these cells predicts the clinical outcome. The α6-subunit is enriched in TICs in breast, prostate and colorectal cancers [44,46,49] as well as squamous cell carcinoma [48] and is important for the maintenance of glioblastoma stem cells [45]. Likewise, the expression of the β4-subunit has been described to serve as a marker for the propagation of lung cancer cells [50]. In melanoma, de novo expression of the αvβ3 integrin has been associated with the ability to convert a non-invasive radial growth phase melanoma into an invasive, vertical growth phase melanoma [51,52,53]. The molecular mechanism involved in the regulation of integrin expression, specifically in invasive tumor cell populations, has been unravelled for example by Kato et al. [54]. The analysis of collective tumor cell migration revealed that β1 integrin expression was restricted to the leading cells (LC), in contrast to the following cells. Interestingly, the LC-specific β1 integrin expression was posttranscriptionally regulated by the tripartite motif-containing 27/myocardin-related transcription factor (TRIM27/MRTF-B) complex in response to the loss of intercellular adhesion [54]. The fact that the activation of β1-subunits triggers the switch from cellular dormancy to metastatic growth in vitro and in vivo identified the β1-subunit-containing integrins as potential targets for therapeutic intervention [55,56,57,58]. Indeed, preclinical studies have revealed that blocking the β1-subunit with monoclonal antibodies (clone AIIB2) effectively reverts the tumorigenic phenotype in MCF-7 human breast cancer xenografts [59].

One common recognition site for some integrin receptors, including αvβ3, αvβ5 α5β1, αIIbβ3, αvβ6, and α3β1 integrins, is the arginine-glycine-asparagine (RGD) sequence, which was first described in fibronectin and is present in many extracellular matrix components [60]. This sequence represents an ontogenetic old and conserved element and peptides containing this integrin recognition sequence were shown to inhibit experimental metastasis of mouse melanoma cells [61,62]. αvβ3 and αvβ5 integrin antagonists significantly reduced the number of metastasis generated by intrasplenic injection of colon cancer cells; however, the growth of the primary tumor was not affected [63]. Additional preclinical studies suggest that the upregulation of αvβ3 and αvβ5 integrins in sprouting vessels is required for angiogenesis [2,4] and a small-molecule inhibitor of both integrins inhibited tumor angiogenesis in different animal models [3]. Thus the use of RGD-mimetic agents was first envisaged as a strategy to block angiogenesis. The RGD-mimetic cilengitide (EMD 121974) blocks the extracellular domain of αvβ3 and αvβ5 integrins from binding their ligands thereby inhibiting the downstream signalling of FAK/Src/Akt and induces apoptosis in endothelial cells and some tumor cells [5]. However, as none of the predefined clinical subgroups showed a benefit from cilengitide in a randomised, open-label, phase 3 trial in patients diagnosed with glioblastoma [64], the future of this synthetic peptide appears to be vague. Nevertheless, using low dose cilengitide to promote rather than inhibit vascularization in order to enhance drug permeability and efficacy has been shown to be an alternative strategy in pre-clinical models, indicating that learning from failed trials can still provide useful information to improve therapeutic strategies [65].

The use of the monoclonal antibodies PF-04605412 (against α5β1) and MEDI-522 (against αvβ3) showed no or only partial responses in patients with advanced solid tumors, respectively [66,67]. Volociximab (M200) is a high-affinity IgG4 chimeric (82% human, 18% murine) monoclonal antibody that specifically binds to α5β1 integrin. A Phase I clinical trial with volociximab in 21 patients with advanced solid malignancies led to one minor response (renal, 7 months) and only one durable stable disease status (melanoma, 14 months) [68]. Despite being well tolerated, volociximab was not efficacious in a phase II, multicenter, single-arm, two-stage study in platinum-resistant, advanced epithelial ovarian or primary peritoneal cancer patients [69]. Remarkably, in contrast to cancer therapy, integrin inhibitors have been a great success in other pathologies, including multiple sclerosis, Crohn′s disease, psoriasis, rheumatoid arthritis and acute coronary syndromes [70,71].

In summary, these disappointing examples clearly indicate the complexity of integrin-signalling, and a full view of the latest insights as to why the strategy of targeting integrin–ECM interactions is non-satisfying has been provided by Demircioglu and Hodivala-Dilke [72]. Considering the recently increasing knowledge in integrin-mediated mechanotransduction it is fair to emphasize that most of preclinical and clinical approaches (Table 2) neglect the contribution of matrix remodeling in integrin-signalling.

## 4. Changing the Tension in Cancer

The dynamic process of extracellular matrix remodeling is the basis for developmental processes and tissue homeostasis. Knowledge on how changes in matrix topology and stiffness can substantially alter intracellular integrin signalling has expanded dramatically in the last five years and is now referred to as “mechanosensing” or “mechanotransduction”. The biomechanical properties of a tissue with regard to its stiffness (measured in pascals, Pa) vary considerably between organs and tissues and especially tumors are often found to be stiffer than the surrounding or healthy tissues [75,76]. In this context it is worthwhile mentioning that traditional tissue culture-treated polystyrene plates are of orders of magnitude stiffer than most soft tissues in the body and may therefore lead to physiologically irrelevant cellular responses.

Despite the findings that the matrix composition, topology and rigidity may regulate a variety of cell behaviors including proliferation, differentiation, migration as well as carcinogenesis and tumor cell invasion, the mechanism underlying the sensing of mechanical cues and subsequent elasticity-triggered pathways is not yet fully clear. The following examples will summarize the recent findings on how changes in the ECM can promote neoplastic transformation and tumor progression.

### 4.1. Stroma-Driven, Integrin-Mediated Neoplastic Transformation

The transformation or conversion of “normal” cells into tumor cells is strongly linked with mutations in genes that regulate cell growth and differentiation. However, of relevance to this chapter is the observation that changes in the stromal compartment (including stromal cells and the ECM) can contribute to this process. For example, fibrotic diseases are characterized by the deposition of excess fibrous connective tissue, mainly collagen and glycosaminoglycans, a high number of inflammatory cells and induced concentration of bioactive soluble factors. The most well characterized pro-fibrotic mediator is TGF-β which mainly mediates cell-cell interactions [77]. Although reports published in the mid-1980s showed that patients with diseases like cystic fibrosis have an increased risk of getting cancer [67,78], we have just started to appreciate the importance of mechano-transduction-induced integrin signalling.

One example for a possible mechanism in integrin-mediated carcinogenesis results from studies of recessive dystrophic epidermolysis bullosa (RDEB) patients. RDEB is a skin fragility disorder caused by mutations in the *COL7A1* gene encoding collagen VII (C7), a component of the anchoring fibrils at the epidermal-dermal adhesion zone. The loss of C7 leads to friction-induced separation of the skin layers and is associated with proneness to tissue injury, highly inflamed skin and heightened TGF-β expression [79]. Interestingly, RDEB patients exhibit at high risk (up to 90%) for developing invasive cutaneous squamous cell carcinoma (cSCC) by the age of 55, and 80% of the patients die of metastatic tumors within 5 years after their first cSCC [79]. The molecular mechanisms underlying the highly aggressive behavior of RDEB-cSCC have long been elusive. Using in vivo and in vitro genetic models for RDEB, we have recently been able to unravel the mechanisms of the dermal contribution to cSCC progression [80]. Increased TGF-β levels within the highly inflamed skin not only facilitate carcinogenesis by increasing the proliferation of pre-malignant keratinocytes and induction of epithelial mesenchymal transition (EMT), but promote the activation of dermal fibroblasts, which in turn produce a stiff, lysyl oxidase (LOX)-crosslinked collagen-rich ECM [80]. The stiffening of the ECM eventually activates β1-subunit-mediated mechano-transduction leading to increased tumor cell survival and migration via FAK- and Akt-mediated signalling axis. These results are in accordance with the observation that cancer-associated fibroblast (CAF)-remodeled ECM alleviates tumor cell invasion [81], indicating that mechano-transduction-induced integrin signalling in stromal cells can promote the establishment of a pro-tumorigenic matrix.

Therefore, inflammation might be a crucial driver of the microenvironmental remodeling and integrin-mediated neoplastic transformation, however, tissue stiffening alone seems to be insufficient to drive carcinoma. Progressive systemic sclerosis, a multisystem disorder with a high associated mortality, exhibits endogenously stiffened skin but the absolute risk of getting cancer is relatively low [82,83].

### 4.2. Matrix-Stiffening Regulates Malignancy by Enhancing Integrin-Dependent Mechano-Transduction

There is increasing evidence that increased matrix rigidity in diverse tissues favors and regulates malignancy. For instance, women with mammographically dense breasts have an increased risk for developing breast cancer [84]. Thus it is not surprising, that clinically, tumors are often found to be stiffer than the surrounding or healthy tissues [75,76]. Interestingly, the use of Atomic Force Microscopy has revealed that the metastatic competency of melanoma cells is associated with increased cell stiffness and increased heterogeneity of stiffness values in the whole cell population [85]. As concentrations of aberrant collagen crosslinks dramatically increase with age, this could, to some extent, provide an explanation as to why aging is the greatest risk factor for developing cancer.

In breast cancer, increased LOX activity, enhanced collagen crosslinking and raising numbers of FAs triggered β1-integrin induced FAK phosphorylation and subsequent tumor cell invasion [86]. Most importantly, the authors noted that neither ECM stiffness nor forced integrin clustering was sufficient to induce mammary tissue invasion in the absence of oncogenic signatures (analyzed in Ha-ras premalignant cells), highlighting the complexity of carcinogenesis and tumor progression [86]. Increased expression of osteopontin (a glycoprotein which is induced in different tumor entities) by hepatocellular carcinoma cells positively correlates with increasing matrix stiffness and has been suggested to be regulated by the β1-subunit induced activation of the glycogen synthase kinase 3β (GSK-3β)/β-catenin pathway [87].

In contrast to the collagen-binding β1 integrin subunit, the β4-subunit, usually found in HDs of epithelial cells, binds preferentially to laminin-332, which is present in high concentrations in the BM (Table 1). As the breakdown of the BM is one of the initial steps in cancer cell invasion, Chaudhuri and co-workers have raised the question as to whether the composition of the cell adjacent matrix is equally important as ECM density [88]. First of all, they confirmed that increasing stiffening of the ECM is sufficient to facilitate a malignant phenotype, which was the result of increased β4-subunit-dependent activation of the PI3K/Rac1 pathway. However, the addition of basement-membrane components completely abrogated this effect, suggesting that mechanical cues can have completely different effects on tumor cell behavior, depending on the ECM composition and the type of the engaged integrin receptor [88].

**Figure 2:**
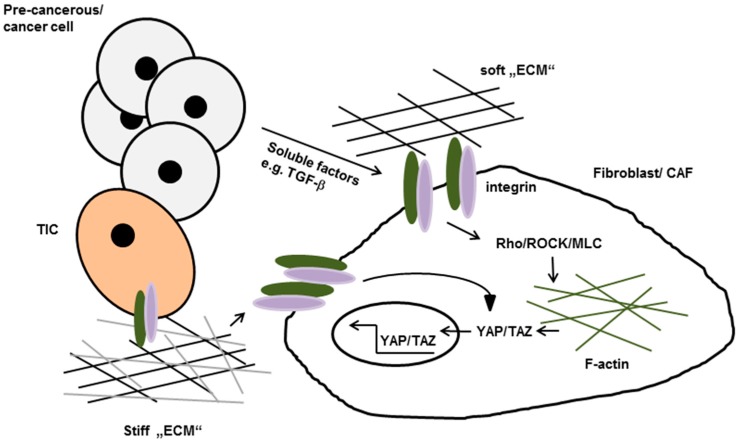
Tumor cell secreted soluble factors promote integrin-mediated activation of Rho/Rho-kinase (ROCK) in normal fibroblasts. Once activated they induces stress fiber formation, increased isometric tension and nuclear localization of the transcription factors YAP and TAZ which lead to the conversion of fibroblasts to cancer-associated fibroblasts (CAFs). Active YAP eventually stabilize actomyosin proteins leading to further matrix stiffening, thereby generating a positive feedback loop. Increases matrix stiffening in turn can promote neoplastic transformation and tumor cell invasion by so called tumor initiating cells (TIC). ECM: extracellular matrix.

The importance of the physical features within the tumor environment raises a question as to the mechanism by which matrix-remodeling is regulated. With regard to this question, Erik Sahai′s research has implicated a mechanosensitive pathway in cancer-associated fibroblasts (CAFs) which is mainly driven by the induced expression of the transcription factor YAP [81]. The enhanced secretion of lysophosphatidic acid (LPA) and TGF-β, supposedly by tumor cells, initially promotes matrix remodeling by stromal fibroblast, a process that requires Rho/ROCK/ Myosin Light Chain 9 (MYL9)/MLC-mediated changes in their contractile actin cytoskeleton. The isometric tension of these fibroblasts enhances according to the increase of matrix-stiffening, which leads to stress fiber formation and integrin-mediated activation of the Src-family kinases at FAs. The increase of Src signalling in turn promotes the nuclear accumulation of YAP and TAZ, followed by increased expression of actin modulating proteins that eventually stabilize actomyosin proteins. This so called “positive feedback loop” further increases matrix stiffening and, as a result of this, facilitates the conversion of stromal fibroblast to CAF cells (see Figure 2) [81]. An additional line of evidence for the importance of the stromal contribution originates from experiments that have been carried out in immune-compromised α11-deficient mice [89]. The growth of A549 lung adenocarcinoma cells and two patient-derived non-small cell lung carcinomas was significantly impeded in the absence of stromal α11-expression. Furthermore, the absence of α11-expression was correlated with decreased collagen reorganization and stiffness of the tumor adjacent matrix, suggesting a potential role of the α11β1 integrin in the conversion of fibroblasts into CAFs [89].

In summary it becomes evident that integrin-mechanotransduction in tumor AND stromal cells are a prerequisite for matrix-stiffening-regulated neoplastic transformation tumor growth and invasion.

### 4.3. Matrix-Stiffening Induced Integrin-Signalling Drives Tumor Therapy Resistance

Despite the overall success of innovative cancer therapies, including targeted and immune checkpoint therapies, both intrinsic and acquired resistance mechanisms still represent a major clinical problem. Thus, tumors that show initial response can rapidly become therapy-tolerant and progress. Unfortunately, drug resistance has turned out to be a multifactorial phenomenon, which is especially based on the property of tumor cells to present high phenotypic cell plasticity and heterogeneity [90,91]. In order to investigate how interactions between cancer cells and the tumor microenvironment affect the response to cancer therapy, some light has been shed on the role of the β1-subunit mechano-transduction and signalling [92,93].

Based on their hypothesis that mechano-chemical changes in the ECM during tumor progression may induce drug resistance in carcinoma, Nguyen and colleagues created a high-throughput drug screening platform based on a poly (ethylene glycol)-phosphorylcholine (PEG-PC) hydrogel system, to test carcinoma cell response to sorafenib as a function of underlying gel stiffness [93]. Surprisingly, sorafenib resistance was increased in conjunction with increased stiffness of the collagen-rich matrix in one liver and three breast cancer cell lines. Furthermore, they identified the β1-subunit, and its downstream effector, JNK, as mediators of tissue stiffening-induced drug resistance, indicating that changes in the rigidity of the underlying substrate can alter integrin-mediated intracellular signalling, which ultimately may change the efficacy of drug treatments [93]. Also, the β1-subunit has been implicated as a driver of drug resistance in erlotinib resistant lung cancer cells [94] as well as in lapatinib and trastuzumab resistance in breast cancer [91,95]. Along the same line, the group of Erik Sahai has discovered a complex and dynamic cross-talk between tumor cells, tumor associated host cells and proteins of the tumor matrix in the context of therapy-resistant BRAF-mutant melanoma cells [92]. Although mutant-specific BRAF kinase inhibitors like vemurafenib or dabrafenib achieve around 50% objective response rate in BRAFV600E-positive individuals, the majority of these patients develop therapy resistance and recurrence after a median duration of ~5–7 months. Hirata et al. recently provided an integrin-dependent, non-cell autonomous mechanism to the BRAF inhibitor PLX4720 and unrevealed the role of the tumor microenvironment in this process [92]. Reactivation of ERK/ mitogen-activated protein kinase (MAPK) signalling was observed in melanoma cells which were localized in areas with high stromal density and this reactivation correlated with resistance towards PLX4720. After re-isolation, cells retained their PLX4720 sensitivity, suggesting that the stiff tumor microenvironment represents a “safe haven” to protect the melanoma cells from drug-treatment. The “paradoxical” activation of melanoma associated fibroblasts (MAFs) has been identified for the enhanced production and remodeling of the tumor microenvironment which subsequently leads to elevated β1-integrin/FAK/Src signalling in resistant melanoma cells [92].

## 5. Conclusions and Future Perspectives

Despite the vast amount of knowledge on integrin biology that has been accumulated in the last three to four decades, we have just started to understand the impact of environmental cues provided by the ECM on integrin-mediated neoplastic transformation and cancer progression. Nevertheless, improving our understanding of ECM remodeling in pathological conditions is crucial for uncovering novel therapeutic targets and treatment strategies. In particular, the increasing number of reports on resistance-mechanisms in tumor-therapy highlights the importance of the development of innovative technologies, as suggested by Nguyen et al., which enable accurate monitoring, imaging and quantification of the ECM [93]. On account of this, the use of three-dimensional cell culture models mimicking diverse rigidity as well as the incorporation of different integrin binding ligands into the high-throughput drug screening process will provide a comprehensive portrayal of the impact of integrin-mediated mechano-transduction and is therefore a prerequisite to a better understanding of the dynamic interaction between tumor cell and the adjacent matrix.

## Figures and Tables

**Table 1:** Ligand specificity of integrins.

Integrin	Prototypic Ligands
α1β1 (CD49a, VLA1)	Collagen IV, I and IX
α2β1 (CD49b, VLA2)	Collagen I, IV and IX
α3β1 (CD49c, VLA3)	Laminin-511, -332, -211
α4β1 (CD49d, VLA4)	Fibronectin, VCAM-1
α5β1 (CD49e, VLA5)	Fibronectin
α6β1 (CD49f, VLA6)	Laminin-511, -332, -111, -411
α7β1	Laminin-511, -211, -411, -111
α8β1	Fibronectin, vitronectin,
α9β1	Tenascin-C, VEGF-C, VEGF-D
α10β1	Collagen I, IV, II and IX
α11β1	Collagen I, IV and IX
α6β4	Laminin-332, -511
αvβ1 (CD51)	Fibronectin, vitronectin
αvβ3	Vitronectin, fibronectin, fibrinogen
αvβ5	Vitronectin
αvβ6	Fibronectin, TGF-β-LAP
αvβ8	Vitronectin, TGF-β-LAP
αEβ7 (CD103, HML-1)	E-cadherin
α4β7	MadCAM-1, fibronectin, VCAM-1
αLβ2 (CD11a)	ICAM-1, -2, -3, -5
αMβ2 (CD11b)	Fibrinogen
αXβ2 (CD11c)	Fibrinogen
αDβ2 (CD11d)	ICAM-3, VCAM-1
αIIBβ3 (CD41)	Fibrinogen, fibronectin

**Table 2:** Selection of integrin inhibitors for cancer therapy in clinical studies. Reports of all clinical trial summary results are published on Clinicaltrials.gov.

Target	Inhibitor	Clinical Trial	Ref.
α5β1	ATN-161 (small peptide antagonist)	Phase II: patients with advanced solid malignancies	[73]
α5β1	Volociximab (mAb)	Phase I: patients with advanced solid malignancies	[68]
α5β1	Volociximab (mAb)	Phase II: patients with therapy-resistant epithelial ovarian cancer and primary peritoneal cancer	[69]
αvβ3, αvβ5	Cilengitide (EMD 121974)	Phase III: glioblastoma patients	[64]
α5β1	PF-04605412 (mAb)	Safety study: advanced solid tumors	[66]
αvβ3	Etaracizumab (MEDI-522)	Phase I: metastatic solid tumors	[74]

mAb = monoclonal antibody.
